# A Free-Operant Reward-Tracking Paradigm to Study Neural Mechanisms and Neurochemical Modulation of Adaptive Behavior in Rats

**DOI:** 10.3390/ijms20123098

**Published:** 2019-06-25

**Authors:** Vanya V. Stoilova, Sina A. Wette, Maik C. Stüttgen

**Affiliations:** Institute of Pathophysiology, University Medical Center of the Johannes Gutenberg University Mainz, 55131 Mainz, Germany; swette@students.uni-mainz.de

**Keywords:** dopamine receptors, muscimol, matching law, operant conditioning, reversal learning, punishment

## Abstract

The ability to respond flexibly to changing environmental circumstances is a hallmark of goal-directed behavior, and compromised flexibility is associated with a wide range of psychiatric conditions in humans, such as addiction and stress-related disorders. To identify neural circuits and transmitter systems implicated in the provision of cognitive flexibility, suitable animal paradigms are needed. Ideally, such models should be easy to implement, allow for rapid task acquisition, provide multiple behavioral readouts, and permit combination with physiological and pharmacological testing and manipulation. Here, we describe a paradigm meeting these requirements and employ it to investigate the neural substrates and neurochemical modulation of adaptive behavior. Water-restricted rats learned to emit operant responses for positive reinforcement (water reward) within minutes in a free-operant conditioning environment. Without further training, animals were able to track changes in the reward schedule. Given prior evidence that the medial prefrontal cortex (mPFC) and the dopaminergic system are required for flexible behavior, we aimed to assess both in more detail. Silencing of mPFC compromised flexible behavior when avoidance of punishment was required. Systemic injections of the D2-receptor agonist quinpirole and the D2-receptor antagonist eticlopride had complex, differential impacts on reward seeking and adaptive behavior.

## 1. Introduction

Cognitive flexibility (CF) is a core executive function generally defined as the capacity to efficiently adjust behavior in response to changing situational demands [1]. In humans, CF confers a higher potential for learning in children [2] and predicts academic achievement in college students [3]. Furthermore, CF facilitates resilience to negative life events and everyday stress among adults [4], and is in turn impaired in adults exposed to early life stress [5]. Compromised CF is a common deficit in many neurological and psychiatric conditions, such as anxiety- and stress-related disorders [6], depression [7], mania [8], ageing [9], autism spectrum disorder [10], anorexia nervosa [11], and obsessive-compulsive disorder (OCD; [12]). Better characterization of the neural substrates and neurochemical modulation underlying CF will therefore not only provide a better understanding of how the brain works to generate flexible behavioral output, but it can also provide insight into the pathophysiology of these disorders and suggest novel treatment approaches.

In animals, different aspects of CF are usually measured by three different behavioral paradigms: Attentional set shifting, the effortful T-maze task, and, most frequently, reversal learning (RL; [13]). Many different implementations of RL exist [14], which all have in common that one out of two or more different stimuli or responses is associated with a specific outcome (usually reward), and that this association is reversed after training. The main dependent variable in these paradigms is the time it takes the animal to adapt its behavior as a consequence of the changed contingency.

RL hinges on many brain areas, including the prefrontal cortex (PFC). Substantial evidence implicates PFC in a set of cognitive (executive) functions (e.g., References [15,16]), such as salience detection and attention, working memory, inhibition and switching, that serve to effectively implement cognitive flexibility and thus to optimize performance in complex environments [17]. Accordingly, intactness of the PFC is required for successful adaptation to changes in the environmental circumstances. In line with this premise, PFC dysfunction has been implicated in clinical disorders, such as schizophrenia, autism, OCD and attention-deficit hyperactivity disorder, which are hallmarked by difficulties to regulate and organize behavior and cognition in a proper way to situational requirements. Moreover, considering the rich dopaminergic input to the PFC, it is believed that perturbation in the dopaminergic neurotransmission may contribute to the pathophysiology of these disorders (e.g., Reference [18]).

Here, we employed a reward-tracking paradigm to study the neural basis of adaptive behavior. The paradigm is easy to implement, allows for rapid task learning, provides multiple behavioral readouts of interest, and allows for combination with physiological recordings and pharmacological manipulation. Although similar in spirit to spatial RL, the task is not trial-based, but instead constitutes a free-operant procedure, in which the animal can continuously act to procure rewards. Free-operant procedures carry the advantage that the continuous stream of behavior is not artificially partitioned into discontinuous segments (trials). As a result, free-operant tasks are more time-efficient because behavior is sampled continuously without the need for an intertrial interval, and we therefore hypothesized on the basis of previous studies [19] that task learning would be extremely fast (1–2 sessions). Moreover, it has repeatedly been argued that behavior under free-operant conditions differs from that in trial-based paradigms, e.g., regarding the sampling of information (for example by visits to a reward location, or by patterns of saccadic eye movements to scan a visual scenery) and changes in the rate of responding, which can be used as a sensitive indicator of confidence or reward expectation in free-operant paradigms only [20,21]. Abandoning a trial-based task structure (although hardly new; [22]) furthermore requires devising fundamentally different kinds of quantitative models of behavioral performance (describing behavior in real time rather than in discrete steps) which has led to important theoretical insights as to the computations being carried out by the brain during task execution [23,24].

We will first describe the task structure and acquisition in detail. Then, we further characterize task performance under conditions of changing reinforcement schedules (requiring adaptive behavior). Last, we demonstrate in two experiments how the task can be combined with pharmacological manipulation of the brain, and that inactivation of the medial prefrontal cortex (mPFC) and alterations in dopaminergic transmission both affect adaptive behavior in a distinctive manner.

## 2. Results

**Figure 1:**
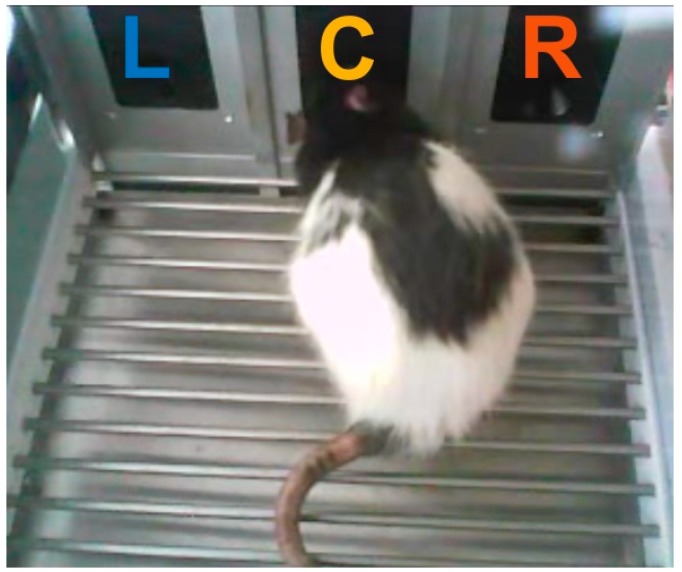
Picture of the operant chamber with a rat poking into the center (reward) port. Reward (30 µL of water) could be triggered by poking into the left (L) and right (R) ports at random intervals. Water reward is delivered at the center (C) port. The stainless steel grid floor was used to deliver mild foot shocks in later experiments.

We adapted, modified, and extended a simple reward-based learning task from Reference [19]. Rats were tested in an operant chamber featuring three conical nose ports arranged next to each other at one wall of the chamber (Figure 1). Rats were required to poke into the left (L) and right (R) ports to trigger reward delivery at the center (C) port. Reward delivery could be elicited by pokes of >0.4 s duration into either the left or the right port on two concurrent RI (random interval) schedules. More specifically, at each port, the reward could be triggered after a random time interval had elapsed (the port is then said to be ‘armed’); the inter-reward time was the mean of the RI schedule, the distribution of inter-reward times is geometric (see Section 4, Materials and Methods, for details). Two concurrent and independent RI schedules were set up for the two operant ports L and R. The optimal strategy to harvest as many rewards as possible in a given session from a given port is to poke at intervals shorter than the minimum RI at that port. To harvest the maximum number of rewards from both ports, animals should distribute their responses such that they visit a port with a lower RI (more frequent arming) more often than a port with a higher RI (less frequent arming). In such situations, animals have been found to allocate their responses such that the relative response proportion for one port approximates the relative reward proportion for that port, a classic finding from experimental psychology known as the ‘Matching Law’ [25]:

R_L_/(R_L_ + R_R_) = Rf_L_/(Rf_L_ + Rf_R_) (1)

In the context of our task, R_L_ is the number of nose pokes allocated to the left nose port, R_R_ is the number of nose pokes allocated to the right nose port, Rf_L_ is the number of reinforcers (water rewards) obtained after leftward choices (nose pokes), and Rf_R_ is the number of reinforcers obtained after rightward nose pokes. In the following, the left-hand side of Equation (1) will be referred to as P(R_L_), the right-hand side will be referred to as P(Rf_L_). Matching behavior is optimal in the sense that it maximizes long-term reward [26,27].

In the following, we describe a series of experiments conducted to establish and characterize task performance, as well as to investigate the degree to which responding is determined by prefrontal and dopaminergic mechanisms. First, we examined how quickly animals acquire the basic structure of the task. Second, we quantified the extent to which the animals exhibit matching behavior in stationary environmental contingencies by running the animals on five different reinforcement schedules, each of which lasted three days. Third, we explored how cognitive flexibility manifested itself as animals were required to track repeated changes in reward rates and rapidly adapt behavior. Fourth, we extended the task to require flexible avoidance of punishment for either R_L_ or R_R_, and tested to what extent the effects of punishment on behavior are altered by transient inactivation of mPFC. Finally, we carried out systemic administration of drugs modulating dopaminergic D2-receptors to examine their involvement in task performance.

### 2.1. Initial Learning

Ideally, rats should pick up the task within a single session, without extended training involving gradual shaping of behavior. To see whether this is feasible we cast six naïve water-restricted rats into the operant chamber and confronted them with the full paradigm.

**Figure 2:**
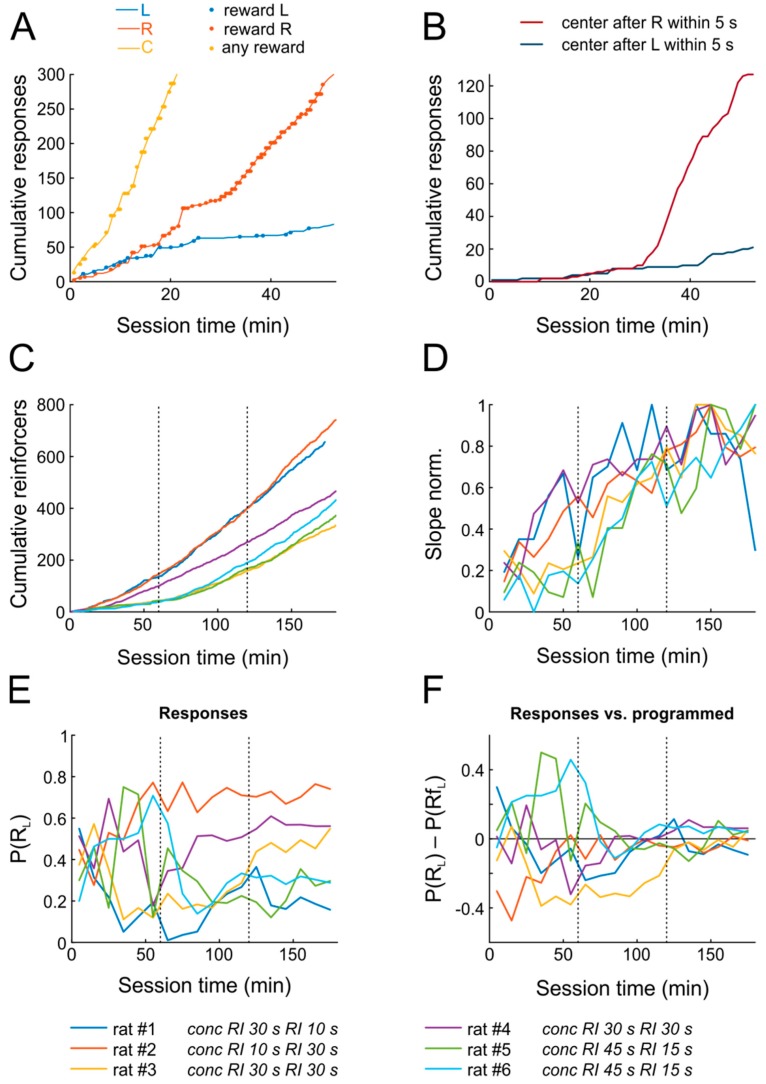
Rats pick up the basic task structure in the very first training session. (**A**) Cumulative numbers of operant responses in 1-min bins (time intervals) for pokes into the left (blue), right (red), and center (reward, yellow) port for one example animal on the first day of training. (**B**) Cumulative responses into the center port within 2 s after left (blue) or right pokes (red) for the same animal. (**C**) Cumulative numbers of reinforcers (in bins of 10 min) for all six rats across the first three sessions (separated by vertical dotted lines). The color code key for panels CDEF is given at the bottom of the figure. (**D**) The number of reinforcers obtained per 10-min bin over the first three days, normalized to the maximum number of reinforcers obtained in any bin by a given rat. For five out of six rats, the maximum number was obtained well before the end of the third session. (**E**) Relative response proportions P(R_L_) for the six rats over the first three days. (**F**) As in E, but after subtraction of programmed relative reinforcement proportions P(Rf_L_) from P(R_L_) to highlight that all animals attained matching (horizontal line, zero difference) by the third day of training.

Figure 2A,B shows the data from a representative animal confronted with the schedule *conc* RI 30 s RI 10 s (i.e., concurrent random-interval schedules for R_L_ (30 s) and R_R_ (10 s); accordingly, the right response port was on average armed three times as often as the left response port). As a result of random explorative behavior, the rat began poking into the operant and reward ports almost immediately after introduction into the chamber, thereby triggering occasional rewards.

Strikingly, within the first training session, the rat not only learned that nose poking yields water reward (as indicated by accelerating response rates), but also developed a clear preference for the right choice port within only 15–20 min, roughly matching its relative response proportion P(R_L_) = R_L_/(R_L_ + R_R_) to the relative reward proportion P(Rf_L_) = Rf_L_/(Rf_L_ + Rf_R_), consistent with the Matching Law (Figure 2A). Similarly, the cumulative frequency of reward port entries following right port pokes increased dramatically 30 min after the onset of the session (Figure 2B), indicating that the animal anticipated the outcome and moved to the center port to retrieve the reward.

Figure 2C,D shows the progression of learning for all six subjects across the first three days. All animals managed to obtain substantial numbers of reinforcements already on the first day. The rate at which reinforcers were obtained increased significantly on the first two days, but not anymore on the third day, indicating that performance reached maximal levels already by the end of the second session, i.e., within less than two hours (*n* = 6; repeated-measures ANOVA across six 10-min bins; day 1: F(5,25) = 2.9, *p* = 0.035; day 2: F(5,25) = 6.1, *p* = 0.0008; day 3: F(5,25) = 1.2, *p* = 0.324).

The six rats were tested with different *conc* RI RI schedules. Following the Matching Law, we would expect each rat to match its P(R_L_) to its specific P(Rf_L_). While rats on the first day exhibited seemingly random preferences for L and R ports (Figure 2E,F), they all closely matched relative response proportions to the programmed relative reinforcement rates by the end of the second day already.

To sum up, animals began poking for reward early after introduction into the chamber, rapidly increased response rates to obtain rewards, and distributed their responses as predicted by the matching law within two sessions only, signifying successful completion of task learning.

### 2.2. Matching Behavior under Steady-State Conditions

After confirming that task acquisition is rapid, we split subjects into two groups. In the first group, we investigated whether the behavior was consistent with the Matching Law under a range of reward contingencies. As is usual in the literature on matching, we kept reinforcement ratios constant for several days, and assessed response rates only on the last (third) day after the contingency was in effect. To that end, three animals were assigned to five different *conc* RI-RI combinations; each combination was run for three consecutive days. The order of the conditions was counterbalanced across subjects (Table 1).

**Figure 3:**
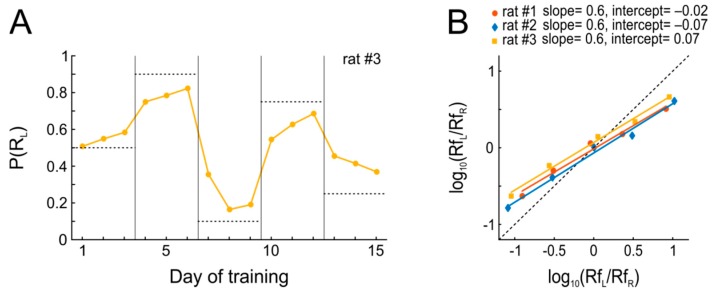
Rats quickly adapt when reinforcement contingencies change every three days. (**A**) Adaptation to new reward contingencies over five blocks. The panel depicts P(R_L_) of an example animal over time. Horizontal dotted lines represent P(R_L_) which would perfectly match P(Rf_L_) within each block. Vertical lines separate blocks of conditions. (**B**) Matching behavior after three days of constant contingencies for all three animals. Regression lines were fitted to the data separately for each animal using Equation (2). Dotted black main diagonal represents the strict matching relation.

The results from this experiment are summarized in Figure 3. Within the three days of each condition during which reward schedules were kept stable, every animal quickly adjusted its P(R_L_) such that it approached P(Rf_L_) (matching). Put differently, the probabilistic performance of the animal adjusts to invariant reward availability, which in turn decreases the variance in behavior and maximizes the overall harvested reinforcement, thus approximating optimal behavior as predicted by the matching law (Figure 3A). However, it is evident from Figure 3B that the rats’ actual behavior systematically deviated from the predictions of Equation (1). Since Equation (1) does not provide a sufficiently good expression of the relations between the available and harvested reward of the actual data, we use another equation known as the “Generalized Matching Law” [28],

log(R_L_/R_R_) = *a* × log(Rf_L_/Rf_R_) + log *c* (2)

where *a* is the slope and *c* is the intercept of the line fitted to the data; when *a* = *c* = 1, Equation (2) reduces to Equation (1) (“strict” matching). Figure 3B shows that the observed behavior departed from strict matching, such that the animals systematically allocated their responses to the more profitable option less often than the matching law would predict (slopes <1, ‘undermatching’), which is rather commonly seen [29]. In contrast, choice bias was close to 1 for all animals, indicative of only mild biases for either of the two ports.

To summarize, we find (1) that animals are able to flexibly adapt to changes in reinforcement schedules without extra training, and (2) that adaptation is mostly achieved within a single behavioral session.

### 2.3. Adaptation to Changing Reward Contingencies

Ideally, a paradigm to study cognitive flexibility in animals should allow one to do so in a single session. However, in trial-based reversal learning and set-shifting tasks with rodents, conditions are only changed from one day to the next [14]. To see whether rats in our paradigm would exhibit rapid adaptation to changes in reinforcement contingencies, we assigned three subjects to a testing sequence in which relative reward proportions changed either every three sessions, once per session, or thrice per session (see Table 2). Throughout testing, session duration was 60 min and schedules alternated between *conc* RI 45 s RI 15 s and *conc* RI 15 s RI 45 s.

**Figure 4:**
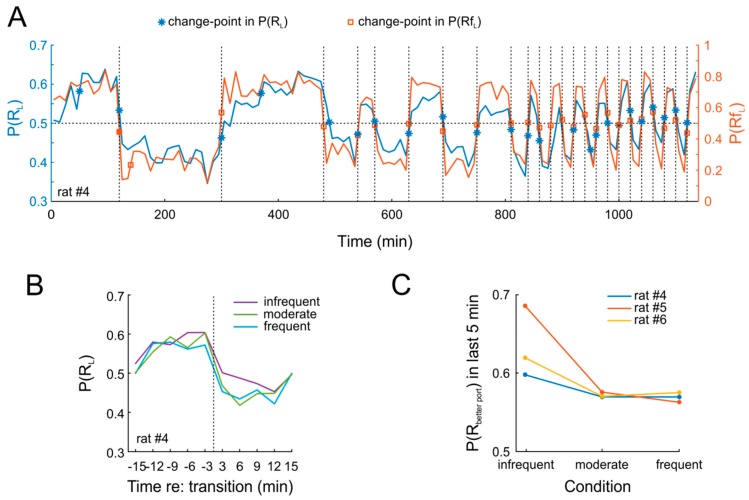
Rats quickly adapt when reinforcement contingencies change multiple times per session. (**A**) Dynamic response allocation resulting from changes in P(Rf_L_) across all 15 experimental sessions in 10-min bins for an example animal. Blue and red symbols denote change-points for responses (asterisks) and reinforcements (squares). Thin horizontal dotted line denotes unbiased responding; vertical dotted lines denote changes in P(Rf_L_). (**B**) Relative choice proportion before and after changes in reinforcement contingency (vertical dotted line) for infrequent (purple), moderately frequent (green), and frequent (cyan) changes. The data from the other rats tested with different sequences were highly similar. (**C**) Comparison of relative response proportions during the last five minutes prior to transitions across all three conditions.

As expected, the animals rapidly adapted behavior in response to the effective P(Rf_L_). Figure 4A shows data from an example session. It is obvious that P(R_L_) closely tracks P(Rf_L_) throughout 19 days of testing. This pattern was highly similar in the two other animals. In order to quantify this finding, we applied a change-point detection algorithm [30] to both P(R_L_) and P(Rf_L_). Due to the near-instantaneous change in response allocation [24], we expected that the algorithm would identify all changes in P(Rf_L_) and, more importantly, would also detect change points in P(R_L_) at the very same time points. This was indeed the case for nearly all switches (Figure 4A). Strikingly, rats tracked changes closely also when they occurred with high frequency (thrice per session, i.e., every 20 min). Importantly, we found no obvious difference in the degree to which animals adapted to the new contingencies, regardless of whether these were in effect for 20 min on one day or for 180 min over three consecutive days (Figure 4B). Nonetheless, adaptation was not complete after 20 min, as indicated by a comparison of P(R_L_) over the last five minutes of each contingency (Figure 4C).

### 2.4. Flexible Adaptation to Punishment and mPFC Involvement

In most of the prevailing literature on adaptive behavior, animals are tested on tasks involving only positive consequences. In contrast, natural environments are challenging in that most action can have both negative and positive consequences. In rare cases, studies employing choice procedures, negative outcomes are indeed incorporated through time-out punishments (e.g., Reference [31]), which however tend to have relatively mild effects even when time-outs last for tens of seconds or even minutes [32]; furthermore, time-out punishments of extended duration may considerable prolong session durations. Foot-shock punishment, in contrast, can be very brief (0.5 s and even less), although great care has to be taken not to completely suppress responding [33].

We wondered whether the task might also lend itself to the study of aversive action outcomes (i.e., punishment). We modified our paradigm such that, in every behavioral session, animals were first confronted with 20–30 min of testing under symmetric reward conditions. Then, the punishment was introduced for responding to a randomly selected choice port under an RI 20 s schedule. The intensity of punishment for each animal was adjusted such that a mild shift in response allocation was observed. We found that shock intensity could be titrated such that it was mildly aversive, as indicated by a measurable, but incomplete reduction of the punished response.

**Figure 5:**
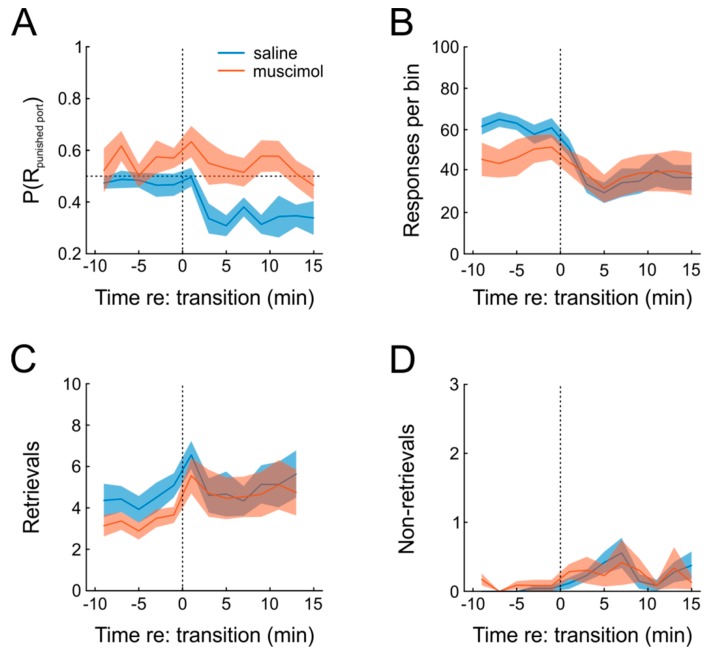
Adaptation to punishment and involvement of mPFC. (**A**) Mean P(R_L_) in consecutive, non-overlapping 2-min bins across animals after saline (blue) or muscimol (red) infusion. P(R_L_) was rectified, such that values <0.5 signify a preference for the unpunished response port. Under saline, the animals exhibited a clear preference for the non-punished option. Vertical dotted line highlights the time of punishment introduction. (**B**) As in (A), but showing operant responses. (**C**) As in (**A**), but showing the number of rewards retrieved per time bin. (**D**) As in (**A**), but showing the number of non-retrieved rewards in all panels. Shading represents SEM.

To investigate the role of the medial prefrontal cortex (mPFC), a structure believed to be involved in adaptive behavior [14], we outfitted the same six rats as used before with guide cannulas for intracerebral substance infusions, targeted to the mPFC of both hemispheres. We hypothesized that transient inactivation of mPFC by local administration of the GABA-A receptor agonist muscimol would prevent rats from adapting their behavior after the introduction of punishment for one of the responses. Figure 5A shows that this was indeed the case. Under saline, animals exhibited a clear increase in preference for the unpunished response, which was absent when muscimol was infused prior to testing, as indicated by significant main effects of both factors drug and time, as well as a significant interaction in a repeated-measures ANOVA (*n* = 6; time: F(12,60) = 3.8, *p* = 0.0003; drug: F(1,5) = 11.9, *p* = 0.018; time × drug: F(12,60) = 2.0, *p* = 0.04). Moreover, introduction of punishment resulted in a slight significant decrease of responding under both saline and muscimol, which was somewhat more pronounced for saline (Figure 5B; repeated-measures ANOVA, time: F(12,60) = 10.3, *p* = 1.4 × 10^−10^; drug: F(1,5) = 1.0, *p* = 0.35, time × drug: F(12,60) = 4.0, *p* = 0.0002).

Another behavioral measure to evaluate the organization of behavior is to count the number of rewards that were actually retrieved, compared to those that were not. Under punishment-free conditions, virtually all rewards were retrieved by the animals (Figure 5C,D). When punishment was introduced, however, animals waived some of the outcomes (thus avoiding punishment, but also missing rewards), but this type of behavior was not affected by mPFC inactivation (repeated-measures ANOVA; time: F(12,60) = 2.6, *p* = 0.007; drug: F(1,5) = 0.02, *p* = 0.89; time × drug: F(12,60) = 0.69, *p* = 0.75). In order to further characterize the effect of mPFC inactivation on the behavioral expression of pain sensation, we tested six different animals on the hot-plate test after both muscimol and saline infusion and found no significant difference in response latency (paired *t*-test; T(5) = 0.9, *p* = 0.40), although the small sample size obviously does not allow a firm statement on this issue.

In sum, we were able to successfully incorporate negative action outcomes into the task. Importantly, punishment-induced response suppression was highly reliable in all animals but incomplete at the same time, allowing us to continuously measure the effects of muscimol and punishment on operant behavior. Additionally, we found that transient inactivation of mPFC abolished behavioral adaptation.

### 2.5. Effects of Systemic Dopamine Receptor Manipulations

As cognitive flexibility has previously been associated with dopaminergic function [14], we next tested whether the dopaminergic system is implicated in task performance. Rats were injected with either the dopamine (DA) D2-receptor agonist quinpirole, the D2-receptor antagonist eticlopride, or vehicle (saline) subcutaneously approximately 20 min before testing. In these experiments, the sessions lasted 60 min and employed a single switch in reinforcement ratios after 30 min from session begins. The animals worked on *conc* RI 22 s RI 90 s (i.e., reward ratios of 4:1, and vice versa) schedules of reinforcement which were maintained for 30 min before RI values were alternated between operant ports. The first schedule on a given day was identical to the second schedule the preceding day, thus, adjustment of response ratios was required only once per session.

**Figure 6:**
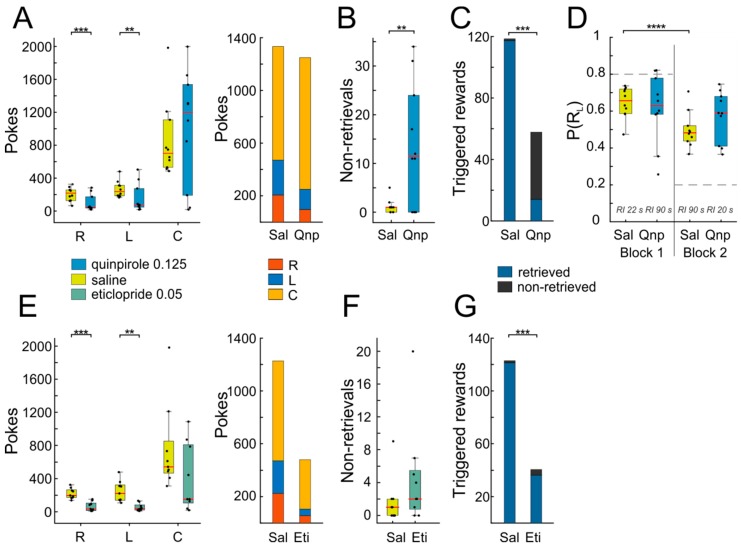
Systemic administration of dopamine D2-receptor compounds alters task performance. (**A**) Left: Quinpirole administration reduced overall responding to both response ports (L and R), but not at the reward port (C). Right: Total number of pokes at the L, R and C ports displayed separately for saline (Sal) and quinpirole (Qnp). (**B**) Quinpirole significantly increased the number of non-retrieved rewards. (**C**) A total number of triggered rewards with those that were retrieved in blue and those that were not retrieved in black. (**D**) P(R_L_) before (Block 1) and after (Block 2) mid-session change in the reward schedule, which required the animals to adapt their responses to the reversed reward ratio as in the first half of the session. Animals changed their responses, according to the reinforcement ratio (indicated in the graph) after saline administration, but not after quinpirole administration. Dashed horizontal lines indicate optimal behavior as predicted by the Matching law. (**E**–**G**) As in **A**–**D**, but for behavior after eticlopride vs. vehicle administration. In all box plots, individual data points (black dots) are laid over a box depicting the 25th and 75th percentiles; the horizontal red mark indicates the median, whiskers extend to the most extreme observations, outliers are plotted individually. Asterisks indicate significant differences between drug and vehicle as determined by paired samples t-tests (** ≤ 0.01, *** ≤ 0.001, **** ≤ 0.0001).

Injection of quinpirole led to a marked suppression of operant behavior, as indicated by reduced nose-poking into both side ports (Figure 6A, *n* = 10, right port: T(9) = 4.89, *p* = 0.0008; left port: T(9) = 3.62, *p* = 0.006; in this and the following analyses, paired *t*-tests were used). Since the animals visited the response ports L and R less frequently after quinpirole administration, they also triggered fewer rewards compared to the saline condition (Figure 6C, T(9) = 4.56, *p* = 0.001). Therefore, one would expect also a decrease in center port poking, as responding is extinguished, due to lack of positive reinforcement. Interestingly however, the average response rate at C was unaffected, The latter finding is even more surprising given that the animals not only failed to produce usual numbers of rewards (as noted above), but they additionally failed to retrieve a considerable fraction of the few triggered rewards (Figure 6B,C; non-retrieved Sal vs. non-retrieved Qnp: T(9) = 3.38, *p* = 0.008). The same effect was observed with a lower dosage of quinpirole (~2/3; data not shown). Additionally, quinpirole administration interfered with adaptive response reallocation after a mid-session reversal in the reward ratios for L and R responses. As shown in Figure 6D, animals changed their responses according to the change in reinforcement ratio from the first to the second block of the session after saline injection, but not after quinpirole injection (Sal block 1 vs. Sal block 2: T(9) = 7.33, *p* = 0.00004; Qnp block 1 vs. Qnp block 2: T(9) = 1, *p* = 0.35). This result indicates that the dopamine D2-receptor is involved in adaptive behavior.

Not unexpectedly, the effects of eticlopride differed from those of quinpirole. While eticlopride also led to a drastic reduction in operant responding (Figure 6E, *n* = 9, right port: T(8) = 4.87, *p* = 0.001; left port: T(8) = 3.8, *p* = 0.005), in turn resulting in low numbers of triggered rewards (Figure 6G, T(8) = 4.8, *p* = 0.001), unlike quinpirole, the number of non-retrieved rewards was not affected (Figure 6F,G, T(8) = 1.35, *p* = 0.21), indicating that responding to the reward signaling cue remained intact.

The effect of eticlopride on task execution was too marked to allow detailed analysis of switching behavior in the middle of the session, precluding us to investigate whether dopamine D2-receptor antagonism is implicated in adaptive reward-tracking.

## 3. Discussion

Our main intention was to implement and characterize a behavioral paradigm built on previous ones [19] that would enable us to study adaptive behavior. We found (1) that rats reached asymptotic operant performance in two to three sessions, (2) that they matched relative response rates to relative reward rates already by the end of the second training session, and (3) that multiple changes in reinforcement contingencies could be carried out per session (in rodents, reversals often take several sessions [14,34]; see Reference [35]).

Rapid task acquisition not only saves experimental time, but will also allow for the tracking of single neurons electrophysiologically during learning, which is often not feasible in operant conditioning which frequently takes several days or weeks, and it is difficult to track the same neurons over more than a few hours with standard electrode arrays designed for chronic implantation. Also, the rapidness of learning will easily allow for an investigation into the effects of certain drugs on the establishment of an operantly conditioned response.

In a similar vein, being able to change reinforcement contingencies multiple times per session allows conducting electrophysiological recordings of single neurons to see how firing changes from before to after the change, and also when conditions are changed back to initial again, as is easily accomplished in monkeys [36] and pigeons [37], but rarely in rodents. Similarly, drug effects are most straightforward to interpret when only a single injection is necessary to observe effects on adaptive behavior.

A critical feature of the task is that it constitutes a free-operant paradigm in which the animal is free to emit responses and consume rewards on a real-time basis. Free-operant tasks do not shoehorn behavior into a trial structure, they are more time-efficient because no intertrial intervals are needed, and it is likely for these reasons that learning progressed so quickly. Moreover, adjustment to a change in reward contingency was observed immediately upon its first introduction, in line with previous suggestions that the reward-tracking behavior we harnessed for our task is unconditioned, i.e., natural behavior [19]. Lastly, although learning and adaptation behavior was characterized in only six animals, the high degree of similarity of the behavioral results (learning speed, adaptation, matching) between animals testifies to the robustness of the paradigm.

A possible future addition to the task would be to construct a cognitive model of reward-tracking under free-operant conditions [24] containing one or two free parameters representing latent variables to be fitted to individual animals’ data with and without pharmacological brain manipulation.

Having established the basic structure of the free-operant reward-tracking task, we explored whether it can be extended to encompass studying how animals change behavior upon introduction of negative outcomes (punishment), an arguably understudied aspect of decision-making (as also pointed out by [38]). One frequently encountered problem in the study of punishment is that behavior can be completely and permanently suppressed when it is too intense [39], while at the same time response suppression is only transient when punishment is too mild ([40]; for classic, but still relevant reviews, see Reference [33,41,42]). Through a gradual increase in shock intensity every few days, we managed to repeatedly produce a significant, but incomplete response suppression, a crucial factor for conducting single-neuron recordings in upcoming experiments. The reliability of response suppression and the high consistency of the effect across rats, along with the paradigm providing a more powerful within-subject statistical comparison [43], enabled us to perform additional pharmacological experiments and to find significant and consistent effects, even with a small sample size of only six animals.

Since the dorsomedial (prelimbic) prefrontal cortex has been implicated in some forms of adaptive behavior [14,35], as well as the processing of pain [44,45] before, we investigated the effects of mPFC inactivation on responding. We found that mPFC inactivation resulted in a slight decrease of responding even before the onset of punishment and prevented the shift of choice allocation away from the punished port, relative to saline controls. What could be the reason that animals fail to adjust their behavior after the introduction of punishment? In principle, there are three explanations.

First, pain sensation could be altered. However, Reference [44] showed that muscimol-induced mPFC inactivation lowered the perceptual threshold for painful thermal stimuli, while leaving the threshold for mechanical pain unaffected. Also, we did not find an increase in response latency under muscimol, although the small sample size prevents a firm conclusion on this issue. A second possibility is that not the sensation itself, but the aversiveness of the pain might be reduced (i.e., the emotional response). Third, it could be that the punishment inflicted pain and emotional stress response during mPFC inactivation just as in control sessions, but that flexible control of responding was impaired. Further experiments using larger samples are required to differentiate these possible explanations.

Stimulating D2-receptors with quinpirole strongly suppressed operant responding, although animals did not exhibit any obvious general deficits in motor activity. These results are consistent with previous reports describing robust suppression of lever press responding for food or water reward after quinpirole administration [46,47]. In contrast, quinpirole did on average not decrease responding to the reward port, arguing against an unspecific suppression of behavior. Moreover, we observed that frequently after obtaining the reward at the center port, most of the animals continued to persistently poke into the reward port beyond the time of reward consumption, indicating that this behavior was dissociated from the ingestion of the water reward, as reported previously [48]. Similarly, quinpirole has been found to induce compulsive checking behavior [49,50]. In line with these findings, medication with DA agonists is related to impulsive and compulsive patterns of goal-directed behaviors, such as pathological gambling, overeating, compulsive shopping, and hypersexuality [12,51,52]. Furthermore, the animals in our study not only persisted in visiting the reward port, but additionally ignored the fact that the majority of these visits did not result in reward delivery, and were even unable to retrieve a substantial fraction of rewards which they did trigger (non-retrievals, Figure 6C), which together would be expected to extinguish poking into the center port. Against this expectation, the animals in our experiment did not show a reduction of responding, indicating that this behavior is resistant to extinction, as has been suggested before [48,53].

It has been demonstrated that the dopamine activity reflects the discrepancy between expected and obtained reward [54,55]. Thus, phasic alterations in dopamine release can serve as a learning signal when the environmental circumstances have changed, and behavior needs to re-adjust to suit new demands. Additionally, reward-paired cues can potentially initiate reward seeking actions by evoking both an increase in phasic mesolimbic dopamine release and sustained elevation in extracellular dopamine concentration [56,57]; similarly, phasic reductions in dopaminergic activity accompany the omission of an expected reward. Quinpirole might blunt dopamine signaling such that, due to sustained postsynaptic D2-receptor activation, rewards are continuously expected, and the absence of these expected rewards is not properly signaled, thereby hampering animals to efficiently adjust behavior in response to the changing demands of the situation and to thereby suppress responses that no longer represent an optimal solution to the problem. The suppression of responding to the operant ports might therefore directly result from increased attention to the reward port.

While eticlopride also suppressed operant responding, it did not increase the number of non-retrieved rewards. Since the animals retrieved the larger proportion of the triggered rewards, it was not possible to directly observe the effect of eticlopride on extinction. However, previous reports showing that the D2-like receptor antagonist sulpiride facilitates learning of extinction [58]. The reduction in operant performance might therefore be interpreted such that the sustained postsynaptic antagonism, due to eticlopride was interpreted as a negative reward prediction error, thereby leading to the extinction of the operant behavior.

In summary, administration of drugs acting on dopamine D2 receptors led to alterations in operant behavior presumably by interfering with the postulated involvement of dopaminergic signaling in reward-related processes and related modulation of cognitive flexibility.

## 4. Materials and Methods

### 4.1. Subjects

Subjects were twelve male Long Evans rats (Charles River, Sulzfeld, Germany). The rats were six weeks old upon arrival in the lab and weighed between 200 and 250 g. All experimental protocols and animal procedures were approved by local authorities (Landesuntersuchungsamt Rheinland-Pfalz, the national investigation office of the state of Rhineland-Palatinate, Germany, permit G14-1-069, issued 2014/10/10). Animals had free access to food, but their water consumption was restricted. During the weekend and on days without experimental sessions, animals were given ad libitum access to water. On days of testing, animals received their daily water ration in the experimental chamber. The weight of the animals, as well as the amount of water reward obtained during the session was controlled on a daily basis, and supplemental water was provided if an animal received less than 8 mL water reward during a single training session. Previous studies showed that rats continuously gain weight under similar water restriction schedules and do not exhibit weight loss or suffer from physiological impairments as assessed by post-mortem organ inspection and hematologic examination [59,60,61]. Rats were housed in individual cages in an air flow cabinet (UniProtect, Zoonlab, Castrop-Rauxel, Germany) which maintained a constant temperature around 23 °C and humidity at >50%. Animals were kept on a 12-h inverted light-dark cycle (lights on at 8 pm). Experiments were carried out at a regular time during the dark phase.

### 4.2. Behavioral Apparatus

Behavioral testing was performed in an operant conditioning test chamber (ENV-008, Med Associates, Georgia, VT, USA) with inner dimensions 48 × 27 × 28 cm (L × W × H), equipped with three nose ports with a conical entry (LIC.80117RM, Lafayette Instrument, Lafayette, IN, USA) and a stainless steel grid rod floor (Figure 1). The operant chamber was enclosed within a sound-attenuating cubicle. Nose ports were located 4 cm above the level of the floor at the front wall and had a diameter of 3 cm and were spaced at intervals of six centimeters. The chamber was constantly illuminated by a house light. A webcam was used for continuous monitoring of behavioral performance. The center port (“reward port”) was used for water reward delivery, and the two side ports (henceforth, “operant ports” or “response ports”) were used as operant response devices. Entries of the nose ports were signaled by a photo beam detector. Water rewards were delivered via silicone tubing into the apertures of the central port from an external peristaltic pump. Behavioral testing and online data collection were controlled by a Windows computer system with in-house software written in Spike2 and a Power1401-3 data acquisition interface (Cambridge Electronic Design Limited, Cambridge, UK). The auditory stimulus used in punishment and pharmacology experiments (1.4-kHz pure tone, 4 s, ~85 dB SPL) was delivered through a speaker attached to the ceiling of the sound-attenuating chamber. Scrambled foot shocks were administered through a stainless-steel grid floor at intensities of 0.27–0.53 mA using a standalone shocker module (ENV-414SA, Med Associates, Georgia, VT, USA). The intensity was adjusted individually for rats.

### 4.3. Behavioral Testing

Rats were handled daily (1–2 h per day) for five days prior to the start of training. The handling involved the experimenter playing with the animals and offering them peanut butter, yogurt, fruits, or nuts. On the first day of training, animals were individually placed into the operant chamber for one hour. Reinforcement (30 µL of water which could be retrieved from the center port) could be obtained by nose-poking for >0.4 s into either the left- or the right-side port. In this session, animals had to learn that some of the pokes to the left and right response ports yielded reward at the center port. Nose-pokes triggered reward delivery on concurrent random-interval (RI) schedules. Two animals were tested on *conc* RI 30 s RI 30 s, another two animals on *conc* RI 15 s RI 45 s, and another two animals on *conc* RI 30 s RI 10 s (counterbalanced as to which operant port was associated with the higher RI value). RIs give expected inter-reinforcement intervals and were implemented such that, once per second, a random number *x* between 0 and 1 was generated. If *x* was smaller than the reciprocal of the RI value in seconds (*p*, i.e., *p* = 1/RI [s]), a reward was automatically delivered at the center port. For example, an RI of 30 s was implemented by setting *p* to 1/30 = 0.0333. This way, the expected inter-reward interval is 1/*p* = 30 s. The distribution of inter-reinforcement intervals for RI schedules varies according to a geometric distribution. Once *x* < *p* at a certain response port (i.e., the port was “armed”), reward delivery at the center port would be triggered by the next nose-poke of the subject with a duration of at least 0.4 s (changeover delay; [25]), regardless of when that nose-poke would occur. The two choice ports were independent, i.e., at any given time either of the ports could be armed, both of them, or none of them. An implication of using RI schedules is that, the longer the subject has not visited a specific port, the higher the probability that it has been armed, and that a reward can be triggered. When two concurrent RIs are implemented, as in the present study, subjects will accordingly visit both response ports. Since both response ports may be armed simultaneously, a subject can collect the maximum possible reward if it repeatedly switches between both options at intervals shorter than the expected RIs.

After three days of training, we changed RI reinforcement ratios for all six subjects. In three rats, we investigated whether animals show matching behavior. For these rats, we kept ratios constant for three consecutive days and then switched to a new contingency. In total, five contingencies were tested over fifteen days (every weekday; Table 1). In the other three rats, we investigated whether subjects could switch to another reinforcement contingency within a short period of time—a hallmark of cognitive flexibility [13,14]. In these animals, we swapped reinforcement contingencies multiple times from *conc* RI 45 s RI 15 s to *conc* RI 15 s RI 45 s, at a rate of from one switch in three days to three switches per day over nineteen days (Table 2).

For the punishment experiment, we tested six animals on a constant reinforcement schedule of *conc* RI 45 s RI 45 s. After 20–30 min, punishment (foot shock, 0.5 s duration, 0.32–0.48 mA intensity) was introduced on an RI 20 s schedule for one of the two responses for another 20 min. In the last 20 min, no punishment was administered. We started out with punishment intensities of around 0.3 mA for all animals (based on previous experiments), but found that it was necessary to occasionally increase intensities in steps of 0.01–0.05 mA to stabilize punishment-induced response suppression. At the same time, it is necessary to keep shock-intensities well below levels that will completely suppress responding (ideally around 50%) to the punished nose port. These shock intensities are well below those commonly used in fear conditioning paradigms (up to 1.5 mA [62]). The degree to which shock intensity had to be titrated to reliably elicit a response suppression depended strongly on the individual animal—for some rats, no or only infrequent adjustments were required, for other animals, it proved more difficult to elicit reliable bias. In any case, pharmacological testing was only conducted when punishment-induced response suppression was stable. Importantly, we took care to avoid systematic biases with respect to muscimol vs. saline sessions (also see Section 4.6 below). Moreover, animals were now required to retrieve the reward from the center port within 5 s after it was triggered at a choice port, and availability of a reward (or a punishment) was signaled by a 5-s pure tone at 1400 Hz.

Lastly, to investigate whether dopamine is implicated in task performance, we tested the same and additional six subjects after injection of either a dopamine D1-receptor agonist (quinpirole) or a D2-receptor antagonist. Before the start of the pharmacological tests, we slightly modified the behavioral procedure. First, we moved the center reward port to the opposite side of the chamber. This was done after we observed that after extensive training (several months during which intracerebral infusions of the GABA-A agonist muscimol into the medial prefrontal cortex were made), animals became insensitive to reward contingency changes and executed seemingly stereotypic action sequences (pokes left–center–right–center–left, etc.). Moving the reward port to the opposite side of the chamber was intended to disrupt habitual behavior and render responding sensitive to reward manipulations again (which was successful). Moreover, in order to signal animals when a visit to the center port was in order, we introduced a tone whenever poking into a side port triggered reinforcement at the center port. After tone onset, animals had four seconds to enter the reward port and to trigger reward delivery. Entering the center port turned off the tone and triggered reward delivery, while poking after tone offset was not rewarded, and the reward was omitted altogether. Within 1–2 sessions, the animals picked up the changed arrangement and successfully harvested more than 90% of the scheduled rewards. Motivated by the observation that animals switch between left and right at a very high frequency, we added another variable that defines the minimum time that a poke triggering a reward can have to the previous poke in the opposite response port (CODOP, 2 s). If the animals switch between the options quicker than determined by CODOP, the clock reset, and no reward is delivered. This makes sure that the animals are not uniformly switching between left and right response ports.

These descriptions apply to the first batch of six rats. A second batch of six rats was trained in a similar way to add to the body of pharmacology data (administration of DA-receptor agonist and antagonist). Behavioral data in Figure 1, Figure 2, Figure 3, Figure 4 and Figure 5 are from the first batch of rats; data from the second batch are contained in Figure 6. Table 3 gives an overview of which rat was used in a certain experiment.

### 4.4. Hot-Plate Test

To assess the effect of mPFC inactivation on pain perception, we employed the hot-plate test (Hot/Cold Plate NG 35100, Ugo-Basile, Gemonio, Italy). Animals were placed on a metal plate which was enclosed in a transparent plastic cylinder to prevent the animal from escaping. The temperature of the plate was set to 50 °C. The dependent variable was the time from placement until the animal either licked its hind paws or jumped. As soon as either of the two responses was observed, the plastic cylinder was removed, and the animal could move off the plate. Every examination was video recorded and scored by impartial observers. Unlike the simpler tail-flick test which is believed to predominantly reflect spinal pain reflexes, the withdrawal responses observed in the hot-plate test reflects supraspinal functioning [63].

### 4.5. Surgical Procedures

Animals were anesthetized via intraperitoneal injection of Medetomidine (0.15 mg kg^−1^), Midazolam (2.0 mg kg^−1^) and Fentanyl (0.005 mg kg^−1^). The fur was shaved off the head, and the animals were placed in the stereotaxic frame (Parallel Rail Stereotaxic Instrument; Stoelting, DublinIreland) and fixed using atraumatic ear bars. To maintain a surgical plane of anesthesia, half of the initial dose was administered subcutaneously after 45 min or as soon as the animal exhibited a reflexive toe pinch-response. The scalp was retracted, and the skull was exposed. Stainless steel guide cannulas for intracranial drug infusion (C315G, PlasticsOne, Roanoke, VA, USA, diameter 0.5 mm) were implanted bilaterally at the following coordinates: Anteroposterior (AP), +3.0 mm from bregma; mediolateral (ML), ± 0.6 mm from the midline, dorsoventral (DV) −3.1 mm below the dural surface. Cannulas were implanted at an outwards angle of 10°. After the cannulas were slowly inserted into the brain, the cannula assembly was fixed with dental cement to five skull screws. Dummy cannulas consisting of a stylet and cap were used as protection. During surgery, the rats received between 10 and 18 mL of isotonic saline and 5% glucose solution as fluid substitution and to maintain circulation and metabolism. After completion of the surgery and administration of analgesics (Carprofen) and antibiotics (Enrofloxacin) the anesthesia was antagonized by a subcutaneously administered mixture of Atipamezole (0.75 mg kg^–1^), Flumazenil (0.2 mg kg^−1^) and Naloxone (0.12 mg kg^−1^). Rats were given 7 to 10 days to recover from surgery before behavioral training recommenced. During recovery, rats were given ad libitum access to food and water and were handled for at least 30 min per day.

### 4.6. Intracerebral Drug Infusions

The GABA-A receptor agonist muscimol (Sigma-Aldrich, St. Louis, MO, USA) was dissolved in 0.9 % saline at a concentration of 1 µg/µL and was stored at −18 °C. Before each infusion, rats were mildly anesthetized with isoflurane. An injection cannula (C315I, PlasticsOne) was connected to a 10-μL Hamilton syringe placed in the syringe holder of an infusion pump (Pump 11 Elite, Harvard Apparatus, Holliston, MA, USA). The injection cannula was inserted into the guide cannula to deliver either saline or muscimol into the mPFC at a flow rate of 0.25 µL/min. After the infusion was complete, the injector cannula was left in place for one additional minute to allow for drug diffusion. Anesthesia was discontinued, and rats were placed in their home cage and tested 45 min after the infusion. Muscimol is known to abolish cortical activity for several hours [64]. Due to the explorative nature of these experiments, we tested different dosages of muscimol (range: 0.35–0.6 µL), but found that the effects were extremely similar. Therefore, we averaged the data across dosages separately for each animal and conducted statistical analyses on that data.

### 4.7. Systemic Drug Administration

The D2-type receptor agonist quinpirole and D2-type receptor antagonist eticlopride hydrochloride (both Sigma Aldrich) were dissolved in physiological 0.9% saline and stored at −4 °C. The dose was chosen based on published experiments, as well as on data from pilot experiments with the same rats. We injected either the dopamine D2-receptor agonist quinpirole (0.125 mg/kg dissolved in 0.5 mL physiological saline), the D2-receptor antagonist eticlopride (0.05 mg/kg, dissolved in 0.5 mL saline), or 0.5 mL vehicle (saline) subcutaneously.

Prior to injection, rats were briefly anesthetized with isoflurane (EZ-B800, World Precision Instruments, Sarasota, FL, USA) and received a subcutaneous injection of either the drug or saline. Approximately 20 min after the injection, the animals were tested on the behavioral task (as in previous studies [65]).

Two rats were excluded from the analysis of eticlopride data, due to the insufficient number of operant responses (less than 20). Another rat had to be excluded from the pharmacology experiments altogether because it exhibited increasing difficulties to perform the task even without drug injection.

### 4.8. Histology

**Figure 7:**
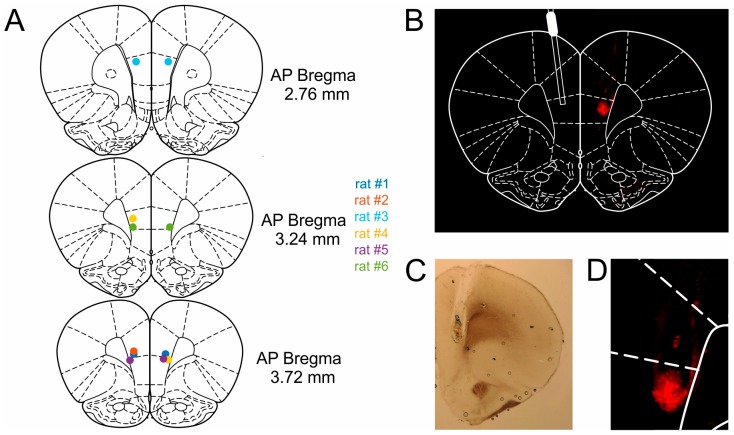
Bilateral microinfusion sites in the mPFC. (**A**) Cannula locations in mPFC, colored dots represent cannula tips of individual rats. Brain diagrams adapted from Reference [66]. (**B**) Coronal slice of the right hemisphere with Evans Blue fluorescence region (right) and schematic representation of the guide cannula (left). The fluorescence is restricted to the prelimbic portion of mPFC. (**C**) Image of a coronal slice under bright field illumination. (**D**) Magnified image of the Evans Blue spread shown in (**B**).

After experiments were completed, the rats were given an infusion of 0.025 % Evans Blue (Sigma Aldrich) in order to verify cannula placement. Approximately 30 min following infusion of the dye, the rats were anesthetized with a mixture of ketamine/xylazine and perfused with 4% paraformaldehyde fixative. Brains were removed and post-fixed in the same fixative for several days, and were subsequently transferred to a 30% sucrose solution for another 2–3 days before slicing. The brains were then frozen and sectioned coronally into 100-µm slices. Prior to mounting onto slides, every other slice was stained with cresyl violet (Nissl), while the other slices were left unstained to better visualize the Evans Blue labeled regions under a fluorescence microscope (Keyence BZ-8000K, Osaka, Japan). All cannula tips were found to be located in mPFC (Figure 7).

### 4.9. Statistical Analysis

The main dependent variables were P(R_L_) and P(Rf_L_). Data were analyzed with paired t-tests and repeated-measures ANOVA. All analyses were performed in Matlab R2018b (The Mathworks, Natick, MA, USA). Change-points were detected using the function findchangepts.m.

## Figures and Tables

**Table 1:** Testing sequence of reinforcement schedules to assess steady-state matching behavior. Each column specifies reinforcement schedules that the respective subject was assigned to during the 15 days of experimentation.

Day	Rat #1	Rat #2	Rat #3
**1–3**	*conc* RI 45 s RI 15 s	*conc* RI 15 s RI 45 s	*conc* RI 30 s RI 30 s
**4–6**	*conc* RI 15 s RI 45 s	*conc* RI 45 s RI 15 s	*conc* RI 5 s RI 55 s
**7–9**	*conc* RI 55 s RI 5 s	*conc* RI 5 s RI 55 s	*conc* RI 55 s RI 5 s
**10–12**	*conc* RI 5 s RI 55 s	*conc* RI 55 s RI 5 s	*conc* RI 15 s RI 45 s
**12–15**	*conc* RI 30 s RI 30 s	*conc* RI 30 s RI 30 s	*conc* RI 45 s RI 15 s

**Table 2:** The sequence of reward rate changes per experimental session for each of three animals. We ran three different blocks of sessions with frequent (three per session) or infrequent (every third day) changes in *conc* RI RI. Numbers in cells thus indicate the number of reward rate switches per day.

Day	1	2	3	4	5	6	7	8	9	10	11	12	13	14	15	16	17	18	19
**rat #4**	0	0	1	0	0	1	0	0	1	1	1	1	1	1	3	3	3	3	3
**rat #5**	3	3	3	3	3	1	1	1	1	1	0	0	1	0	0	1	0	0	1
**rat #6**	1	1	1	1	1	3	3	3	3	3	0	0	1	0	0	1	0	0	1

**Table 3:** Assignment of rats to experiments. (+) the animal participated in the experiment, (±), the animal participated, but data were excluded from the analysis.

Experiment/Rat #	1	2	3	4	5	6	7	8	9	10	11	12
Initial Learning (*n* = 6)	+	+	+	+	+	+						
Matching (*n* = 3)	+	+	+									
Adaptation (*n* = 3)				+	+	+						
Punishment (*n* = 6)	+	+	+	+	+	+						
Quinpirole (*n* = 10)	+	+		+	+	+	±	+	+	+	+	+
Eticlopride (*n* = 9)	+	+		+	+	+	±	±	+	+	+	+
Hote-Plate Test (*n* = 6)							+	+	+	+	+	+

## Abbreviations

C	center
CF	cognitive flexibility
CODOP	changeover delay after other-port response
*conc* RI x s RI y s	concurrent random-interval random interval schedule at with a mean of x seconds inter-reinforcement times at the left choice port and a mean of y seconds at the right
D2	D2-receptor
DA	dopamine
dB SPL	decibel Sound Pressure Level
Eti	eticlopride
GABA	gamma-aminobutyric acid
GABA-A	GABA receptor type A
Hz	Hertz
L	left
mPFC	medial prefrontal cortex
OCD	obsessive-compulsive disorder
PFC	Prefrontal cortex
P(R_L_), P(RfL)	relative rate of operant L responses and relative rate of reinforcements from L responses, respectively
Qnp	quinpirole
R	right
RI	random interval
Rf_L_, Rf_R_	reinforcement (water delivery at the center port) triggered by nose-poking into the left and right choice port, respectively
*R_L_*, *R_R_*	responses by nose-poking into the left and right choice port, respectively
Sal	saline
